# A Finger-Shaped Tactile Sensor for Fabric Surfaces Evaluation by 2-Dimensional Active Sliding Touch

**DOI:** 10.3390/s140304899

**Published:** 2014-03-11

**Authors:** Haihua Hu, Yezhen Han, Aiguo Song, Shanguang Chen, Chunhui Wang, Zheng Wang

**Affiliations:** 1 School of Instrument Science and Engineering, Southeast University, Nanjing 210096, China; E-Mails: huhaihuadongda@hotmail.com (H.H.); 220122600@seu.edu.cn (Y.H.); 2 National Key Laboratory of Human Factors Engineering, Beijing 100094, China; E-Mails: shanguang_chen_hf@163.com (S.C.); Chunhui_wang_hf@126.com (C.W.); zheng_wang_hf@yeah.net (Z.W.)

**Keywords:** tactile sensor, surface texture measurement, fabric texture classification

## Abstract

Sliding tactile perception is a basic function for human beings to determine the mechanical properties of object surfaces and recognize materials. Imitating this process, this paper proposes a novel finger-shaped tactile sensor based on a thin piezoelectric polyvinylidene fluoride (PVDF) film for surface texture measurement. A parallelogram mechanism is designed to ensure that the sensor applies a constant contact force perpendicular to the object surface, and a 2-dimensional movable mechanical structure is utilized to generate the relative motion at a certain speed between the sensor and the object surface. By controlling the 2-dimensional motion of the finger-shaped sensor along the object surface, small height/depth variation of surface texture changes the output charge of PVDF film then surface texture can be measured. In this paper, the finger-shaped tactile sensor is used to evaluate and classify five different kinds of linen. Fast Fourier Transformation (FFT) is utilized to get original attribute data of surface in the frequency domain, and principal component analysis (PCA) is used to compress the attribute data and extract feature information. Finally, low dimensional features are classified by Support Vector Machine (SVM). The experimental results show that this finger-shaped tactile sensor is effective and high accurate for discriminating the five textures.

## 1. Introduction

Tactile sensation which means feeling objects by touch is one of the most important factors to sense the environment and obtain information. Klatzky, a professor of psychology, experimentally confirmed that vision has a higher spatial resolution than touch, which can quickly and accurately get very fine details, while tactile sensation on exploring and distinguishing material properties is much better than vision [1]. Thus, studying on tactile sensors which can detect the surfaces of objects and extract features of materials is very valuable. In virtual reality, such as a digital museum project, shapes and textures of the cultural artifact are important to archive and represent haptic impression [2]. In the field of robotics, intelligent robots with tactile sensors can be used to provide more information about surface texture and assist in object recognition tasks [3,4]. In the textile field, tactile sensors are useful for evaluating fabric surfaces because extracting and comparing fabric surface properties accurately can lead to a more stable textile product quality and reduce the costs of production and inspection [5].

In surface metrology, the size of texture elements varies with the purpose of analysis. Coarse texture usually gives the spatial sense and fine texture gives a vibration sense [6,7]. In many cases, they both exist, especially in soft fabrics which surface can be deformed by external force. Thus, perception of the surface texture is a complex process. Designing effective tactile sensors with the capability of extracting features of surfaces is still a challenge task [8].

In previous designs, handheld devices with a probe were a common type of tactile sensor for surface property detection. Pai *et al.* designed a wireless device for haptic texture interaction by simultaneously measuring contact force and acceleration in a handheld probe [9]. Ye *et al.* developed a pen-type texture sensor with strain gauges, PVDF module, force sensor and contact probe to discriminate surface roughness [8], but this measurement method is more suitable for stiff surfaces. The probe tip may deform or hurt soft surface like woven textiles. Computer vision techniques based on cameras or microscopes [10,11] are another good way to explore surfaces using a non-contact method to get fine details. However, they can only reflect spatial information but not mechanical properties and is hard to achieve real-time classification.

As mentioned above, soft surface detection is a relatively challenging problem. In the textile field, Fabric Assurance by Simple Testing (FAST) [12] and Kawabata Evaluation System for Fabrics (KES-FB) [13] are well-known systems for evaluating fabrics. In these systems, samples are cut from fabrics and then mounted on a series of instruments to measure the mechanical properties of the fabrics. Therefore, these complex systems are more suitable for laboratory than industrial measurements.

In recent literature, some new methods and materials are involved in tactile sensors. Muhammad *et al.* designed a MEMS based biomimetic tactile sensor to measure force ranges encountered during tactile exploration of surfaces [14]. Kumar *et al.* developed a flexible tactile sensor based on a thin polydimethylsiloxane (PDMS) film for contact traction distribution [15]. These research projects are still in the early stages and only partial static contact information of surface can be detected.

However, psychophysical research shows that the tactile perception of human finger is not a static process but an active process influenced by both the finger motion and the force applied on the object surface, which includes three steps [16–18]. Firstly, finger touches the surface of object with a slight pressure and repeats a back-and-forth motion at speeds from 2 to 10 cm/s. The particles from the surface texture activate the cutaneous sensory neurons. Secondly, the sensory neurons transduce the physical stimulus into electric signals and then send them to the brain cortex through the spinal cord, brain stem, and thalamus. Thirdly, the cerebral cortex resolves the perceived information and produces results in a specific texture feeling. Smith *et al.* studied the human surface exploration process by the fingertips, and showed the mean normal contact force exerted by humans on surfaces is 1.54 ± 0.50 N [19].

By imitating the human active tactile perception process, we have designed a texture sensor with a simple rotational mechanical structure in our previous research [20,21]. The limitation of the texture sensor is that a piece of sample must be cut from the measured objects and it can only measure in a fixed circular path with rotational motion. In order to extend the detection range to a two-dimensional plane, this paper proposes an improved finger-shaped tactile sensor installed on a mechanical structure with two-dimensional motion. Moreover, samples needn't be cut from the measured objects. This finger-shaped tactile sensor is designed using a PVDF film as sensitive element, which has high piezoelectric effect and unique physical properties to fabricate a high-speed-response and high-accuracy device. We use FFT operation to get original attribute data of the surface in the frequency domain. PCA is utilized to compress the attribute data and extract feature information. Finally, low dimensional features are classified by SVM.

The rest of the paper is organized as follows: Section 2 describes the principle of surface measurement by the PVDF film. Section 3 introduces the design of the finger-shaped sensor. Section 4 describes the mathematical principles of PCA and SVM. Section 5 shows the experiments and results of fabric classification. Finally, Section 6 provides some conclusions.

## 2. Principle of Surface Measurement by PVDF

PVDF is a polymer consisting of long molecular chains with repeated –CF_2_–CH_2_– units. It has strong piezo-pyroelectric activity. Compared with other piezoelectric materials, PVDF is flexible, light, tenacious and inexpensive. Therefore, PVDF film is very suitable for tactile sensor fabrication [22]. The piezoelectric coefficient matrix of PVDF form is usually expressed as [20,21,23]:

(1) $$d_{ij}=\left[\begin{array}{cccccc} 0 & 0 & 0 & 0 & d_{15} & 0\\ 0 & 0 & 0 & d_{24} & 0 & 0\\ d_{31} & d_{32} & d_{33} & 0 & 0 & 0 \end{array}\right]$$

**Figure 1.**
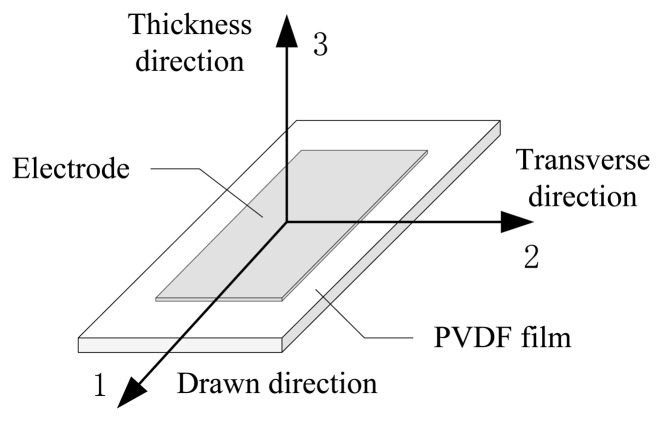
Schematic picture of a PVDF film.

Figure 1 is schematic picture of a PVDF film. The sensitivity of the PVDF film depends on the direction of measurement. The coefficients *d*_31_, *d*_32_, *d*_33_ are the piezoelectric strain coefficient of the direction 1, 2, 3, respectively and −*d*_33_ ≥ *d*_31_ > *d*_32_ > 0. The coefficients *d*_24_, *d*_15_ are the piezoelectric shearing strain coefficient of the film sides in the direction 1, 2, respectively. For the commercially available PVDF film, the piezoelectric shearing strain coefficients are smaller than the piezoelectric strain coefficients about two orders of magnitude.

When a force is applied on the PVDF film, the output charge in the electroded area of the PVDF film is expressed as:

(2) $$\frac{Q}{A_{3}}=d_{31}\frac{F_{1}}{A_{1}}+d_{32}\frac{F_{2}}{A_{2}}+d_{33}\frac{F_{3}}{A_{3}}=d_{31}\sigma_{1}+d_{32}\sigma_{2}+d_{33}\sigma_{3}$$

where *Q* is the output charge, *A*_3_ is the electrode area of the PVDF film, *A*_1_ and *A*_2_ are the cross-sectional areas of the film perpendicular to the *A*_3_. *F*_1_, *F*_2_, *F*_3_ are the applied force in the direction 1, 2, 3, respectively, and *σ*_1_, *σ*_2_ and *σ*_3_ are the tensile stress in the direction 1, 2, 3, respectively. Moreover, the electrode film to which the PVDF film is glued is usually rigid in the horizontal direction, which means *σ*_1_ = *σ*_2_ = 0. Thus, Equation (2) can be rewritten as:

(3) $$\frac{Q}{A_{3}}=d_{33}\sigma_{3}$$

During the sliding movement, a micro-unit of PVDF film will be compressed by the fine particles on the surface, resulting in an amount of charge. Thus, we can get the surface texture characteristics by measuring the output charges of the PVDF film. Usually, the electrode fully covers the surface of the PVDF film that means the electrode area *A*_3_ equals the surface area of PVDF film *Ω*. Let *σ*(*x*,*y*,*t*) denote the strain of point (*x*,*y*) at time *t* on the surface of the PVDF film. When the PVDF texture sensor is connected to the amplifier circuits, the leakage current *i* should be considered. Thus the output charge of the PVDF texture sensor caused by the strain change can be expressed as:

(4) $$\left\{ \begin{array}{l} Q(t)=Q(t_{0})+\underset{\Omega }{∯}(\int_{0}^{t}d_{33}\frac{\partial \sigma_{3}(x,y,t)}{\partial t}dt)\text{dxdy}-\int_{0}^{t}\text{idt}\\ i\approx \frac{Q(t)}{R\cdot C} \end{array}\right.$$

where *Q*(*t*_0_) is the output charge of the PVDF texture sensor at the initial time *t*_0_, *R* is the amplifier equivalent resistance, and *C* is defined as the equivalent capacitance of the PVDF film. Assuming the strain is uniform on the sensitive area of the PVDF film and that the initial output charge *Q*(*t*_0_) = 0. Then the total electric charge *Q*(*t*) induced at each surface electrode of the PVDF film by the strain at the contact area can be given as:

(5) $$Q(t)=\Omega \cdot d_{33}\int_{0}^{t}\frac{\partial \sigma_{3}(x,y,t)}{\partial t}dt-\int_{0}^{t}\frac{Q(t)}{RC}dt$$

When strain *σ*_3_ is changed as a step function, the solution of Equation (5) is:

(6) $$Q(t)=\Omega \cdot d_{33}\cdot e^{-\frac{t}{RC}}\cdot U(t)=Q_{\Omega }e^{-\frac{t}{T}}U(t)$$

where *U*(*t*) is the step function, *T* = *RC* is the response time constant, and *Q_Ω_* = *Ω*· *d*_33_ is the amplitude of output charge caused by strain *σ*_3_ in the electrode area. Equation (6) illustrates that the output charge of the PVDF texture sensor will exponentially decrease with time *t* owing to the leakage current of the sensor and the amplifier. This implies that the texture sensor using the PVDF film as the sensing element is unable to measure the static force but is sensitive to the dynamic force, which is similar to the perceptual characteristic of human fingers.

## 3. The Design of the Finger-Shaped Sensor

### 3.1. Mechanical Design of Finger-Shaped Tactile Sensor Based on PVDF Film

**Figure 2.**
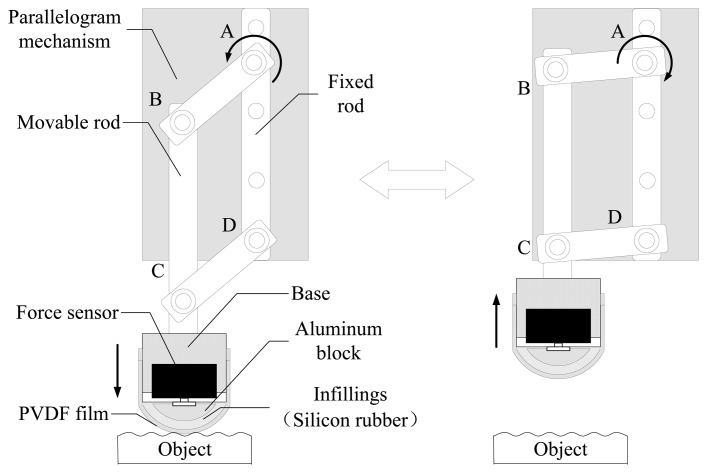
Structure of the finger-shaped tactile sensor.

The mechanical structure of the finger-shaped tactile sensor is shown in Figure 2. The sensor is composed of PVDF film, infillings, aluminum block, force sensor, base and parallelogram mechanism. The base is a cylindrical shape connected to the movable rod of the parallelogram mechanism. The force sensor is fixed to the base and a curved aluminum block is mounted at the end of the force sensor. The curved aluminum block plays a supporting role like our finger bone so that the sensor tip is arched and won't damage soft surfaces. Silicon rubber is used as infilling to fill the cavity between the force sensor and PVDF film, which is capable of transferring the contact force from the PVDF film to the force sensor owing to its soft human tissue-like properties. The arched surface of the silicon rubber is covered with a latex membrane as a protective layer. Then the PVDF film is glued to the latex membrane surface. The PVDF film is 10 mm width, 30 mm length and 30 μm thick. The coefficient *d*_33_ is 21 pC/N. The function of the PVDF film is to measure the strain variation corresponding to the surface texture change when the relative sliding motion between the sensor and the object surface happens.

The parallelogram mechanism is designed to ensure that the sensor is pressed perpendicularly to the surface of the object. It has three movable rods and one fixed rod connected to the motor shaft in point A of Figure 2. When the motor shaft rotates anticlockwise, the sensor will go down slowly to contact the surface along the vertical direction. Meanwhile, the contact force is detected by the installed force sensor. As soon as the contact force reaches a certain value, the motor will stop. Then the contact force between the sensor and the surface won't be changed owing to self-locking function of the motor. Here, we use a FSG1500g touch force sensor manufactured by the Honeywell Corporation (Morristown, NJ, USA) to measure the normal contact force between the PVDF film and the surface of objects. Its measuring range is 15 N with a measurement precision of 0.5% F.S., and its resistance to overload reaches 55 N.

### 3.2. Design of Measurement System

**Figure 3.**
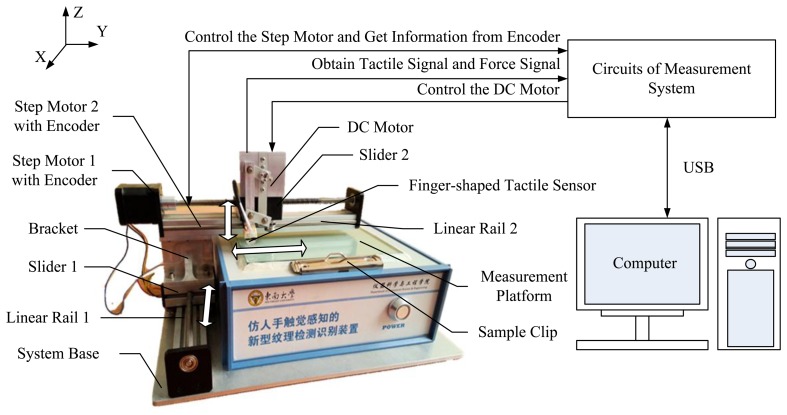
The schematic of the measurement system.

Figure 3 shows the schematic of the measurement system. It consists of a finger-shaped tactile sensor, two-dimensional motion mechanism, measurement platform with two sample clips, system base and the measurement system circuits.

The finger-shaped tactile sensor is installed on the two-dimensional motion mechanism, which is composed of two linear rails with sliders. Linear rail 1 is fixed on the system base to produce movement in the X-direction. Linear rail 2 is perpendicular to linear rail 1 and move in Y-direction. Bracket is the mechanical connection of linear rail 1 and linear rail 2. Each linear rail is driven by a step motor with encoder which can record displacements and speeds. The measurement platform is also fixed on the system base under linear rail 2. Samples are mounted on the platform with sample clips on both sides of the platform and they needn't be cut from the measured objects. Therefore, the finger-shaped tactile sensor can slide both in the X-direction and Y-direction on any part of a sample at a certain speed.

**Figure 4.**
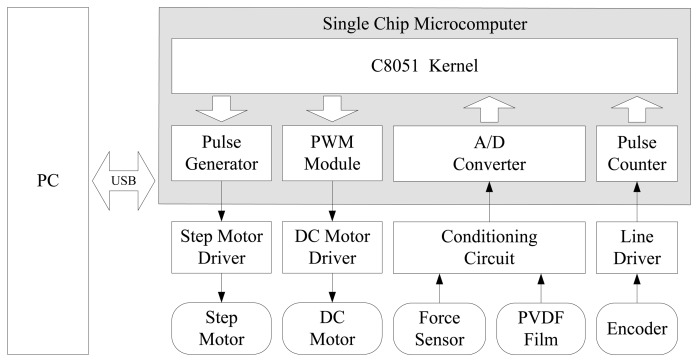
Circuit architecture of the measurement system.

The circuit architecture of the measurement system is given in Figure 4. It includes conditioning circuit for the PVDF film output charge signal and force sensor output voltage signal, DC motor driver, step motor driver, line driver for encoder and single chip microcomputer connected to the computer via a USB interface. When measuring the object surface texture, the computer sends an instruction to the single chip microcomputer and then the single chip microcomputer controls the step motors through step motor driver so that the two-dimensional motion mechanism can take the tactile sensor to a specified position of samples. Then, a PWM signal with a certain duty cycle is generated by the PWM module to control the DC motor, which can adaptively adjust the contact force between the tactile sensor and surface of samples. Force signal is obtained from the force sensor through the conditioning circuit. When the tactile sensor is sliding on the surface of samples, the texture property can be obtained by acquiring the output charge of PVDF film. Encoders are installed on the step motors to measure the rotation angle so that we can obtain the speeds and displacements of sliders.

## 4. Data Dimension Reduction and Classification

### 4.1. PCA-Based Data Dimension Reduction

A Fast Fourier Transformation (FFT) operation is utilized to get the original attribute data of surface in the frequency domain. Then texture signal can be obtained as 12,288 data points in this domain. If the texture signal is used directly for classification, it will require a large amount of calculations, so Principal Component Analysis (PCA) is introduced for dimension reduction.

PCA is a classical and powerful dimension reduction technique. It uses orthogonal transformation to convert the original correlated data points into a set of linearly uncorrelated variables called principal components [24,25]. Suppose there is a matrix *X* with *n* rows and *m* columns that each row represents an *m*-dimensional vector of one sample. Then matrix *X̄*, which the empirical mean of the distribution has been subtracted from the original data set, can be calculated as follows:

(7) $$\begin{array}{cc} \bar{X_{i}}=X_{i}-\frac{1}{m}\overset{m}{\underset{k=1}{\text{∑}}}x_{i}(k), & i=1,2,\cdots ,n \end{array}$$

where $\bar{X_{i}}$ is the *i*-th row of *X̄*. The covariance matrix of *X̄* is:

(8) $$\text{Cov}=\frac{1}{m}{\bar{X}}^{T}\bar{X}$$

Apparently, the matrix *Cov* is a diagonal matrix. As a result, the *m* orthogonal unit eigenvectors, which are the principal components, can be written as follows:

(9) $$E=\left(\begin{array}{cccc} e_{1} & e_{2} & \cdots & e_{m} \end{array}\right)$$

Assuming that the eigenvalues of them are *λ*_1_ ≥ *λ*_2_ ≥ … ≥ *λ*_m_ ≥ 0, the cumulative contribution rate *u_k_* is calculated as follows:

(10) $$\begin{array}{cc} u_{k}=\frac{\overset{k}{\underset{l=0}{\text{∑}}}\lambda_{l}}{\overset{m}{\underset{j=0}{\text{∑}}}\lambda_{j}}, & k\leq m \end{array}$$

then the orthogonal transformation matrix *P* is:

(11) $$P=(\begin{array}{cccc} e_{1} & e_{2} & \cdots & e_{k}) \end{array}$$

The matrix *S*, which consists of the data with *k* dimensions, is calculated as follows:

(12) S=XP

Each row of *S* is the dimension reduction result of corresponding vector in *X*. Thus the original *m*-dimensional vectors can be reduced to *k* dimensions.

### 4.2. Classification Based on SVM

Support Vector Machine (SVM) is a popular supervised learning method to analyze data and recognize patterns in classification and regression analysis. It can maximize the geometric margins and minimize the empirical classification error simultaneously [26,27]. While using SVM in classification, a good separation can be achieved by constructing a hyperplane which separates the classes with the largest margin. Besides linear classification, SVM can efficiently perform a non-liner classification by mapping input vectors into high-dimensional feature spaces.

Suppose there are *n* samples in the training data corresponding to two classes. Each sample includes a vector *S_i_*, (*i* = 1,2,…,*n*). This input vector *S_i_* is mapped into a high dimensional space *H* by applying kernel trick. Guassian radial basis function (RBF), a common choice of kernel, is used in this study [28]. It can be defined as follows:

(13) $$K(S_{i},S_{j})=\exp\left(-\frac{{\left\|S_{i}-S_{j}\right\|}^{2}}{2\sigma^{2}}\right)$$

**Figure 5.**
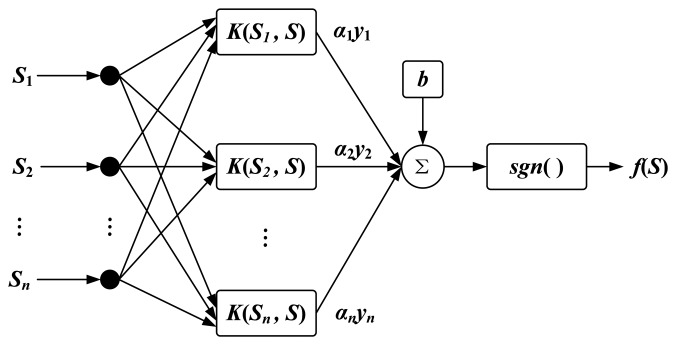
The mathematical form of *f*(*S*).

The training data is used to determine the classification function *f*(*S*). As shown in Figure 5, the mathematical form of *f*(*S*) is similar to a three-layer feedforward artificial neural network.

The function is defined in terms of kernels:

(14) $$f(S)=\mathrm{sgn}\left[\overset{n}{\underset{i=1}{\text{∑}}}{\text{α}}_{i}y_{i}K(S_{i},S)+b\right]$$

where *K* is the kernel function, *b* is a bias term. *y_i_* is the class label with value +1 or −1 and *α_i_* is the Lagrange multiplier coefficient obtained by solving the Quadratic Programming Problem (QPP). However, SVM may not find a separating hyperplane for some data sets such as mislabeled samples. The soft margin SVM [27] by introducing slack variables is more useful for finding hyperplane which splits the samples in feature space. Thus, finding coefficients *α_i_* is equivalent mathematically to maximize:

(15) $$\max Q(\alpha )=\overset{n}{\underset{i=1}{\text{∑}}}\alpha_{i}-\frac{1}{2}\overset{n}{\underset{i=1}{\text{∑}}}\overset{n}{\underset{j=1}{\text{∑}}}\alpha_{i}\alpha_{j}y_{i}y_{j}K(S_{i},S_{j})$$

with the constraints of:

(16) $$\{\begin{array}{cc} \begin{array}{c} \overset{n}{\underset{i=1}{\text{∑}}}\alpha_{i}y_{i}=0\\ 0\leq \alpha_{i}\leq C \end{array} & \left(i=1,2,\cdots ,n\right) \end{array}$$

where *C* is a non-negative regularization parameter used to control the trade-off between maximizing the margin and minimizing the error.

In this study, there are more than two kinds of samples for classification. Therefore, a binary classification method above is not enough to distinguish samples. Multiclass SVM needs to be built. The approach for doing this is to reduce the single multiclass problem into multiple binary classification problems. Binary classifiers should be built to discriminate between every two classes [29]. The “Max Wins” strategy is utilized in this approach [30]. Every classifier assigns the sample to one of the two classes and the assigned class adds one vote. Finally the class with the most votes determines the sample classification. In this way, if there are *N* kinds of samples in classification, (*N* − 1)*N*/2 classifiers are needed in multiclass SVM.

## 5. Experimental Results

### 5.1. Two Dimensional Texture Measurement

**Figure 6.**
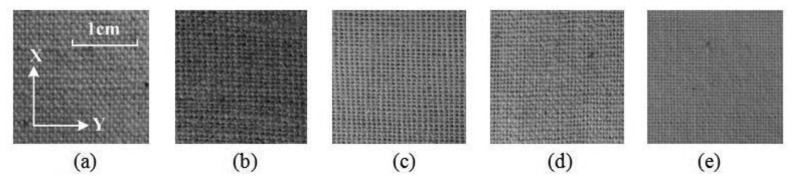
The photograph of five types of linen. (**a**) linen No.1; (**b**) linen No.2; (**c**) linen No.3; (**d**) linen No.4; (**e**) linen No.5.

In our experiment, we chose five samples of linen for texture measurement. Each sample is similar in material but different in yarn density, yarn thickness and weave pattern, which have great impact on tactile sensation [31]. Sixty places are selected randomly on each sample for tactile signal acquisition and 300 groups of data are obtained in total. Figure 6 shows photographs of the five types of linen.

**Figure 7.**
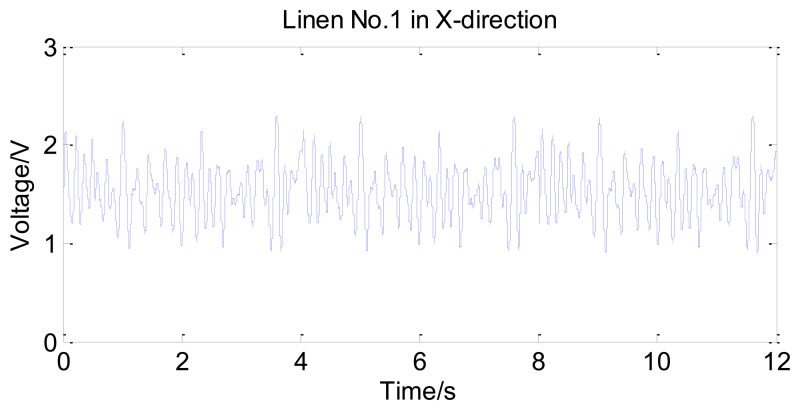
The raw data of linen No.1 in the time domain.

As shown in Figure 6a, some fabrics have different properties in the X-direction and Y-direction. Humans can easily distinguish between the two directions by tactile sensation. Thus tactile signals in different directions are valuable in evaluation. To get comprehensive surface mechanical properties, tactile signals are acquired in both directions during the experiment. The contact force of the tactile sensor applied on the sample of linen is set to be 1.5 N, which is within the fingertip touch force range of 1.54 ± 0.50 N. The sliding speed is set to be 2.55 cm/s, which is ensured by the encoder installed on the step motor. For instance, Figure 7 shows the raw data of linen No.1 in the time domain, which is acquired in the X-direction.

**Figure 8.**
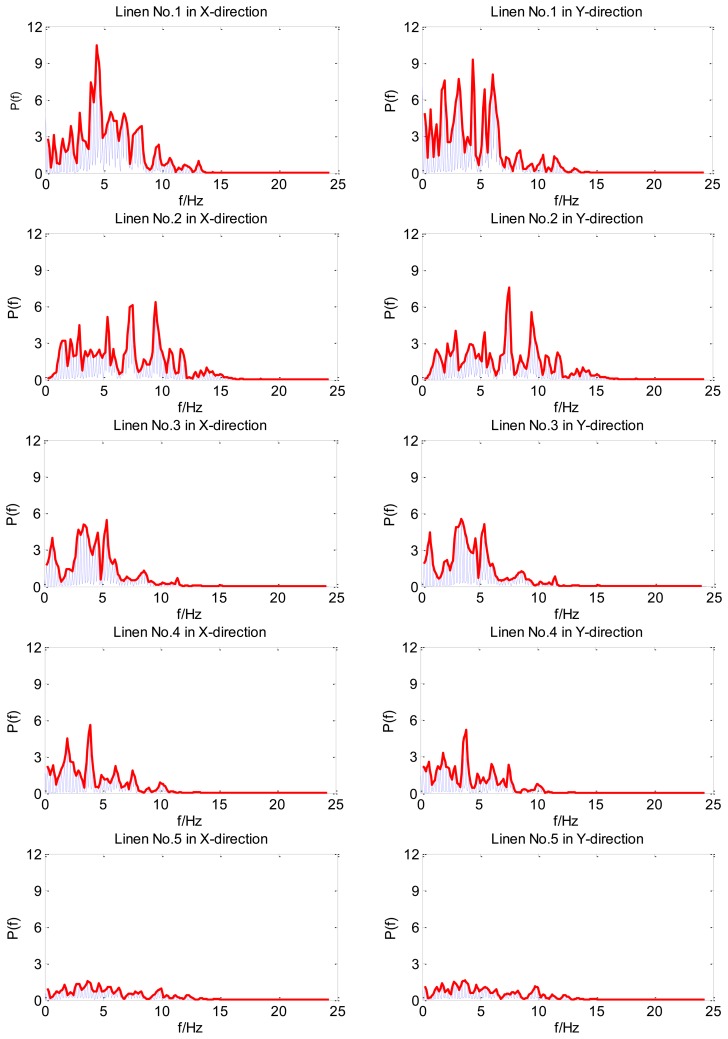
The power spectrum density of five types of linen.

Figure 8 displays the power spectrum density of the five types of linen. The left charts show texture information in the X-direction, and the right charts in the Y-direction. The thick line is the power spectrum density envelope, which can facilitate observation of spectral peaks. As shown in the figure, the surface mechanical features of linen No. 1 are different in the X-direction and Y-direction. However, the remaining four kinds of linen show similar mechanical features in the X-direction and Y-direction, which indicates that their woven structures of the two directions have little difference. Meanwhile, each kind of linen has its own features in the frequency domain. Thus, imitating the motion of human finger, this finger-shaped tactile sensor is capable of detecting in two directions.

### 5.2. Classification Results

**Figure 9.**
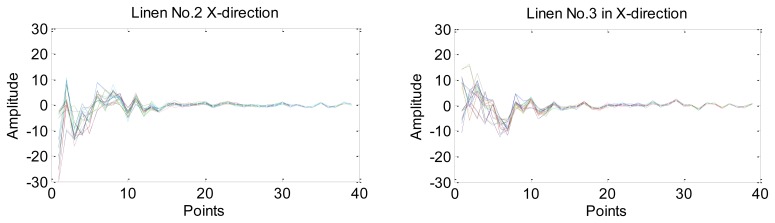
The dimension reduction results of linen No.2 and linen No.3.

We use PCA to reduce the dimension of features in the frequency domain. Taking linens No. 2 and No. 3 for instance, each of them has 60 groups of data in the X-direction, so the matrix *X* can be 60 rows and 12,288 columns, which means *n* = 60 and *m* = 12,288. Then the dimension reduction results can be calculated using Equations (7) to (12). To make *u_k_* > 99%, *k* is set to be 39. Eventually, the 12,288 points can be reduced to a 39 dimensional vector of each sample. Figure 9 shows the dimension reduction results of linen No. 2 and linen No. 3. Lines with different colors represent different groups of data and each figure has 60 curves in total.

As illustrated above, the dimension of features is effectively reduced to 39. In classification, the input vectors of each sample are combinations of the dimension reduction results in X-direction and Y-direction. Thus each input vector is 78-dimensional.

We use SVM to classify the five kinds of fabrics with 78-dimensional features and ten binary classifiers are built for the multiclass SVM. For each type of linen, sixty samples are acquired, in other words, 300 samples in total. We randomly choose 200 samples as training data and the rest are the testing data. All the classification results are shown in Table 1. There is no misclassification for linen No. 1 due to the distinct characteristics of direction. However, linen No. 3 and line No. 4 are sometimes mistaken for each other because they have some similar features in the frequency domain and some noises during the detection lead to more difficulties in their distinction.

Although the five kinds of linen are similar in material and the differences between them are relatively small, their extracted texture features can be classified accurately using PCA and SVM algorithm. As a whole, the accuracy of linen classification reaches 92.0%, which indicates that the finger-shaped tactile senor is effective for fabric evaluation.

## 6. Conclusions

In this paper, we have developed a novel finger-shaped tactile sensor for evaluating fabric surfaces by imitating the human active touching process. A thin PVDF film is used as the sensitive element in the tactile sensor, so that height/depth variation of surface texture can be measured by relative motion with a constant contact force between the sensor and the 2-dimensional surface. Before classification, PCA is used for dimension reduction and the dimension of features is effectively reduced to a 39-dimensional vector. Finally, a SVM method based on the RBF kernel is used for fabric classification. In the experiments, five kinds of linen are used for classification. The accuracy of linen classification reaches 92.0%. The measurement of this proposed sensor is accurate and cost-effective, and in addition samples needn't to be cut from the measured fabrics, making the finger-shaped sensor suitable for fabric quality evaluation and control in the industrial field.

## Figures and Tables

**Table 1.** The results of classification with SVM

**Linen number**	**Number of learning**	**Number of errors/Number in the test set**	**Classification accuracy**
1	40	0/20	100.0%
2	39	1/21	95.2%
3	40	3/20	85.0%
4	38	3/22	86.4%
5	43	1/17	94.1%

Total	200	8/100	92.0%

## References

[1] Roberta L.K., Susan L. (1987). There's more to touch than meets the eye: The salience of object attributes for haptics with and without vision. J. Exp. Psychol. General.

[2] Wataru W., Katsuhito A., Masaharu I., Hiromi T.T. A realtime and direct-touch interaction system for the 3D cultural artifact exhibition.

[3] Mazid A.M., Russell R.A. A robotic opto-tactile sensor for assessing object surface texture.

[4] Nawid J., Claude S. (2011). Majority voting: Material classification by tactile sensing using surface texture. IEEE Trans. Robot..

[5] Ryo K., Kenta N., Motoji Y. (2012). Finger-mounted tactile sensor for evaluating surfaces. J Robot. Mechatron..

[6] Hollins M., Risner S.R. (2000). Evidence for the duplex theory of tactile texture perception. Percept. Psychophys..

[7] Hollins M., Bensmaïa S.J., Roy E.A. (2002). Vibrotaction and texture perception. Behav. Brain Res..

[8] Ye X.M., Byungjune C., Sungchul K., Hyouk R.C. (2010). Profile-based roughness discrimination with pen-type texture sensor. Int. J. Control Autom. Syst..

[9] Pai D.K., Rizun P. The WHaT: A wireless haptic texture sensor.

[10] Ajay K. (2008). Computer-vision-based fabric defect detection: A survey. IEEE Trans. Ind. Electron..

[11] Xin W., Georganas N.D., Petriu E.M. (2011). Fabric texture analysis using computer vision techniques. IEEE Trans. Instrum. Meas..

[12] DeBoos A., Tester D. (2005). Effect of Mechanical and Physical Properties on Fabric Hand.

[13] Kawabata S. (1980). The Standardization and Analysis of Hand Evaluation.

[14] Muhammad H.B., Oddo C.M., Beccai L., Adams M.J., Carrozza M.C., Hukins D.W., Ward M.C. Development of a biomimetic MEMS based capacitive tactile sensor.

[15] Kumar S., Gang L., Mandayam A.S. Flexible membrane tactile sensor for contact traction distribution measurement on a microscale.

[16] Ramona F., Francesco M., Eric C., Jean P.C., Yves B. (2012). Contact of a finger on rigid surfaces and textiles: Friction coefficient and induced vibrations. Tribol. Lett..

[17] Pasquero J., Hayword V. STReSS: A practical tactile display system with one millimeter spatial resolution and 700 Hz refresh rate.

[18] Drewing K., Kaim L. (2009). Haptic shape perception from force and position signals varies with exploratory movement direction and the exploring finger. Atten. Percept. Psychophys..

[19] Smith A.M., Basile G., Theriault-Groom J., Fortier-Poisson P., Campin G., Hayward V. (2010). Roughness of simulated surfaces examined with a haptic tool: Effects of spatial period, friction, and resistance amplitude. Exp. Brain Res..

[20] Song A., Han Y., Hu H., Tian L., Wu J. (2013). Active perception-based haptic texture sensor. Sens. Mater..

[21] Song A., Han Y., Hu H., Li J. (2013). A Novel Texture Sensor for Fabric Texture Measurement and Classification. IEEE Trans. Instrum. Meas..

[22] Qasaimeh M.A., Sokhanvar S., Dargahi J., Kahrizi M. (2009). PVDF-based microfabricated tactile sensor for minimally invasive surgery. J. Microelectromech. Syst..

[23] Dargahi J. (2000). A piezoelectric tactile sensor with three sensing elements for robotic, endoscopic and prosthetic applications. Sens. Actuators A Phys..

[24] Chitradevi N., Palanisamy V., Baskaran K., Aswini D. Designing an efficient PCA based data model for wireless sensor networks.

[25] Zhang Y., Bingham C.M., Gallimore M., Yang Z., Chen J. Applied sensor fault detection and validation using transposed input data PCA and ANNs.

[26] Corinna C., Vladimir V. (1995). Support-vector networks. Mach. Learn..

[27] Vladimir V. (2000). The Nature of Statistical Learning Theory.

[28] Nashat S., Abdullah A., Abdullah M.Z. (2014). Machine vision for crack inspection of biscuits featuring pyramid detection scheme. J. Food Eng..

[29] Levinger P., Lai D.T.H., Begg R., Webster K., Feller J., Gilleard W. The application of multiclass SVM to the detection of knee pathologies using kinetic data: A preliminary study.

[30] Hsu C.-W., Lin C.-J. (2002). A comparison of methods for multiclass support vector machines. IEEE Trans. Neural Netw..

[31] Mine A. (2013). The effect of fabric balance and fabric cover on surface roughness of polyester fabrics. Fibers Polym..

